# The Suprachoroidal Route in Glaucoma Surgery

**DOI:** 10.5005/jp-journals-10008-1197

**Published:** 2016-05-12

**Authors:** Anthony Gigon, Tarek Shaarawy

**Affiliations:** Medical Student (Final Year), Department of Ophthalmology, Geneva University Hospital Geneva, Switzerland; LecturerDepartment of Ophthalmology, Geneva University Hospital Geneva, Switzerland

**Keywords:** Glaucoma surgery, Microinvasive, Review, Suprachoroidal.

## Abstract

Glaucoma surgeries targeting the uveoscleral drainage pathways have been drawing more attention lately. Among all the available techniques, procedures focusing on the supra-choroidal space seem particularly promising, by making use of a presumably efficient and secure outflow route and avoiding subconjunctival filtration blebs.

The purpose of this review is to assess the efficacy and the security of the different suprachoroidal drainage implants, namely the CyPass Micro-Stent, the iStent Supra, the SOLX Gold Shunt, the Aquashunt, and the STARflo Glaucoma Implant.

Most clinical studies seem to currently point toward the direction that there are actual benefits in suprachoroidal surgeries by avoiding bleb-related complications. Nevertheless, even suprachoroidal implants may be subject to scarring and failure. More data are still needed, especially concerning long-term effects, although the approach does seem appealing.

**How to cite this article:** Gigon A, Shaarawy T. The Suprachoroidal Route in Glaucoma Surgery. J Curr Glaucoma Pract 2016;10(1): 13-20.

## INTRODUCTION

Recent times have seen greater attention to glaucoma surgeries targeting the uveoscleral drainage pathways. While subconjunctival drainage implants have been better studied and used for a longer time, complications inherent to the *ab-externo* approach, as well as bleb-related complications might make one wonder if it really is the optimal solution. Thus, the suprachoroidal space, untapped and less well-known, has garnered interest for its supposedly safer approach, as a bleb-less glaucoma surgery. As a consequence, there has been a surge of interest in the development of devices targeting the suprachoroidal space.

Our focus here is to review the different suprachoroidal implants available to date in the market. To do this, we first recapitulate the relevant physiology, pathology, and pharmacology of the suprachoroidal space and the uveoscleral outflow routes. We, then, attempt to evaluate the current situation looking at the evidence concerning the safety and efficacy of each product.

## PHYSIOLOGY

### Production of Aqueous Humor

There are two distinct mechanisms by which aqueous humor is produced. The first is passive ultrafiltration of plasma from blood capillaries. Under physiological conditions, however, it is highly improbable that the latter plays a significant role.^1^

The second mechanism is active, owing to the secretion of solutes by the ciliary epithelium, creating a driving force for water to follow. A key enzyme in this active production of aqueous humor is the carbonic anhydrase, which is targeted by pharmacological agents, such as acetazolamide, used to treat various glauco-matous conditions. The vascular tone is subject to auto-regulation accommodating to hydrostatic pressure, via the adrenergic and cholinergic systems. Stimulation by alpha 1 and 2 agonists induces vasoconstriction, which itself leads to a decrease in aqueous humor production.

### Elimination of Aqueous Humor

Traditional Pathway

As the traditional pathway of aqueous humor drainage is not our focus here, we will briefly mention it without going into details.

Aqueous humor flows from the anterior chamber to the episcleral veins, through the trabecular meshwork and Schlemm’s canal. The trabecular meshwork is anatomically divided into three portions. The uveoscleral portion, which is made of loose collagen and elastin fibers, the corneoscleral portion, and the juxtacanalicular portion, directly in apposition to Schlemm’s canal. The latter is composed of denser fibers and plays a significant role in ocular hypertension, as it is a source of outflow resistance. Beyond the trabecular meshwork is Schlemm’s canal, gathering aqueous humor. From there, aqueous humor is evacuated through collecting channels discharging in the episcleral venous plexus.^1^

Uveoscleral Pathway

The other way of elimination of aqueous humor is the uveoscleral pathway. The lack of epithelium between the anterior chamber and the ciliary muscle allows aqueous humor to flow between the ciliary muscle bundles to reach the suprachoroidal space.^2^ From there, it is then eliminated through the sclera. This outflow is guided by the pressure gradient that exists between the anterior chamber and the suprachoroidal space.^3^

The ciliary muscle constitutes an important factor determining the outflow resistance and changes of its state of contraction directly influence the total uveoscleral flow. Relaxation of the ciliary muscle by atropine increases the uveoscleral outflow and conversely, its contraction by pilocarpine reduces it.^4^

Concerning its importance, the uveoscleral outflow has traditionally been accounted for less than 15% of the total elimination pathway.^5^ However, since then it has been shown that this fraction was in fact much more important than what was initially thought.^6^ Furthermore, age has also been demonstrated to play a significant role.^6^ Toris et al indeed found that uveoscleral outflow accounted for 54 and 46%, in their first group (20-30 years old) and second group (60 years and older) respectively.

## PATHOLOGY

### Ocular Hypertension

In ocular hypertensive patients, aqueous humor dynamics are modified. There is evidence that the proportion of uveoscleral outflow is reduced, constituting only 25% of the total.^7^

### Glaucoma

In glaucomatous patients under maximal medical therapy, the uveoscleral outflow is on the contrary elevated.^8^ It has been hypothesised that in the initial state of the disease a reduction in both the traditional and the uveoscleral pathways were responsible for the intraocular pressure (IOP) elevation, as was observed in ocular hypertensive patients. With the progression of the disease though there would be a redirection of the aqueous humor outflow from the trabecular pathway to the uveoscleral pathway, explaining the high uveoscleral filtration fraction found in glaucomatous eyes.

## MEDICAL THERAPY

### Prostaglandins

The decrease in IOP induced by prostaglandins has two components: an early and direct effect of prostaglandins, and a late long-term effect. The early effect occurs after a single dose of prostaglandin is instilled and is the consequence of the relaxing effect on the ciliary muscle. This was deduced because the forced contraction of the muscle with pilocarpine also prevented the early IOP decrease of prostaglandins.^9,10^

The long-term effect is still not fully understood, but current theories state the implication of matrix remodeling. A reduction of matrix components, such as collagen following application of prostaglandins and prostaglandin analogs was found.^11,12^ The activation of the FP receptor^13,14^ increases matrix metalloproteinases expression which is responsible for the collagen degradation.^15^

In clinical practice, the most commonly used pros-taglandins and prostaglandin analogs are latanoprost, bimatoprost, travoprost, tafluprost and unoprostone.^16^ The most effective drugs were found to be bimatoprost, travoprost, and latanoprost, achieving an IOP reduction of 28 to 31%.^16,17^

## SURGICAL THERAPY

### Ab-interno

CyPass

**Fig. 1:**
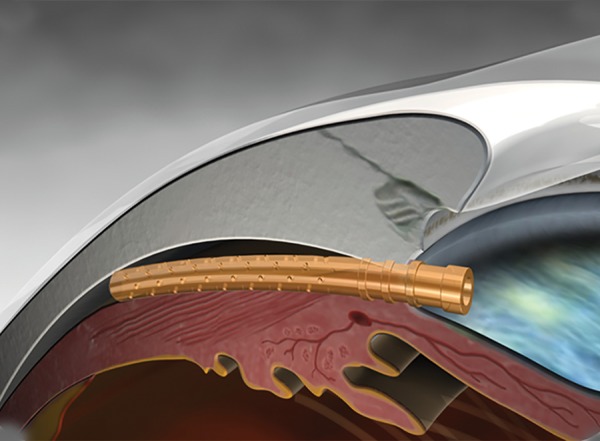
Schematic view of the CyPass Micro-Stent in the suprachoroidal space

*Device:* The CyPass Micro-Stent (Transcend Medical, Inc.) is designed to be implanted in the supraciliary space with or without cataract surgery,^35^ providing outflow of aqueous humor to the suprachoroidal space (Fig. 1). It is currently still an investigational device in the United States but has a CE mark in Europe.

**Fig. 2:**
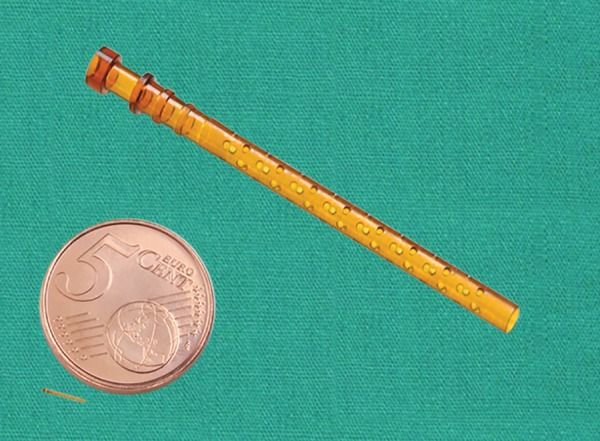
Relative size of the stent

The device has a length of 6.35 mm and an external diameter of 510 μm^18^ (Fig. 2).

**Fig. 3:**
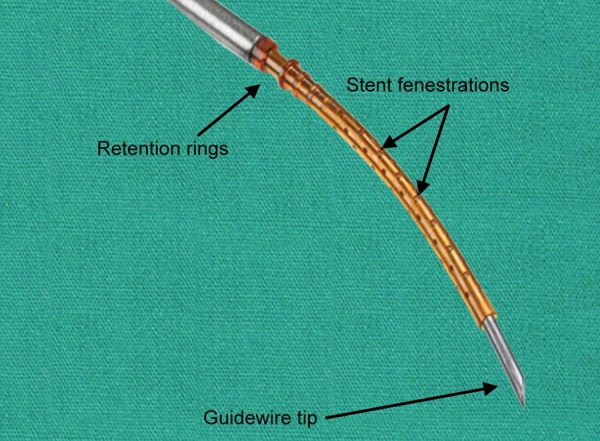
Schematic view of the stent mounted on its guide

It has a slight curvature allowing it to follow the natural sclera shape and once placed in the supraciliary space, retention rings located at the anterior chamber end help stabilizing the Micro-Stent (Fig. 3). The stent also displays various fenestrations along its length to allow overall better filtration (Fig. 3).

*Surgical technique:* This device is designed to be implanted from an *ab-interno* approach, therefore, preserving the conjunctiva.

**Fig. 4:**
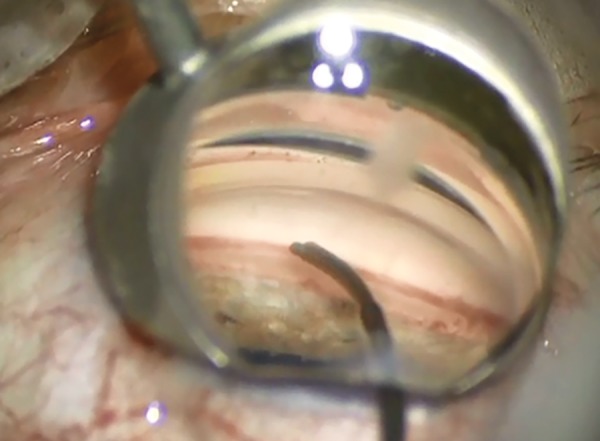
Guide mounted with the CyPass Micro-Stent

**Fig. 5:**
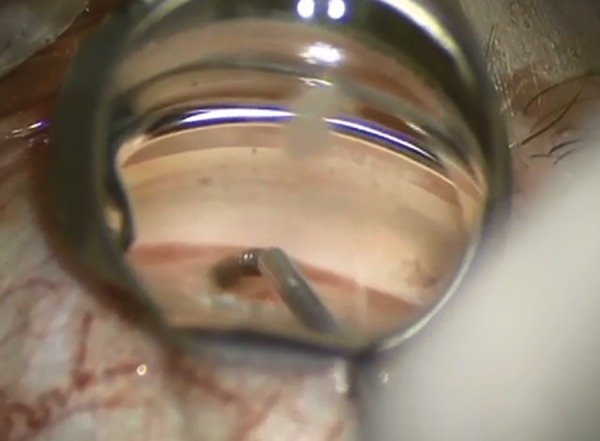
Removal of guide wire, after inserting the implant

**Fig. 6:**
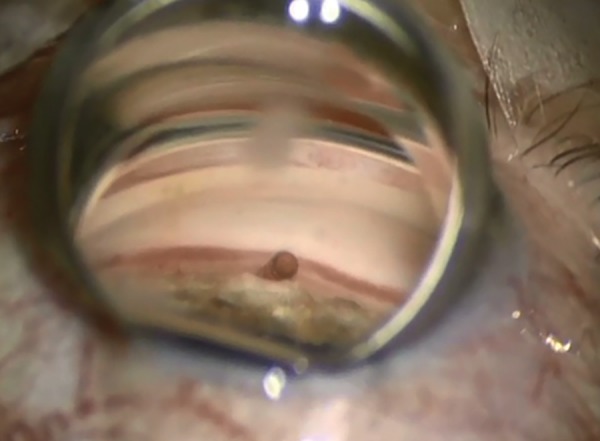
Micro-Stent in place

Miosis is obtained pharmacologically to allow better iridocorneal angle visualization under gonioscopy. For the same purpose, the anterior chamber is filled with an ophthalmic viscosurgical device. A guide mounted with the CyPass Micro-Stent is then passed through a corneal incision. As the iridocorneal angle is reached, gonioscopy is necessary and the tip of the guide is used to dissect the ciliary body from the scleral spur, sparing both structures (Figs 4 to 6). Once the implant is in place and anchored thanks to its retention rings, the guide can be removed.

*Clinical data:* In the United States, the large randomized control trial, COMPASS^36^ is evaluating the device and is estimated to end in 2015. However, to date there are already clinical trials assessing the safety and efficacy of the device^18-21^ So far, studies have shown predominantly positive results.

The primary outcome was always to assess the safety of the device in terms of adverse events. No severe adverse events were noted,^18-21^ but the most common complications included transient early hypo-tony,^18,19,21^ transient IOP increases,^18,19^ and Micro-Stent obstruction.^21^

Secondary outcomes were the evaluation of the effectiveness of the device in terms of IOP reduction and IOP-lowering medication reduction. Two cohorts were evaluated each time, one for patients with initial IOP ≥21 mm Hg prior to surgery and one with IOP < 21 mm Hg. In cohort 1, the IOP reduction ranged from 35 to 37%^19-21^ and the medication reduction from 45 to 55%.^18,20^ In cohort 2, only medication reduction was looked at, since the IOP was < 21 mm Hg to begin with. The medication reduction ranged from 71 to 75%.^18,19^

iStent Supra

**Fig. 7:**
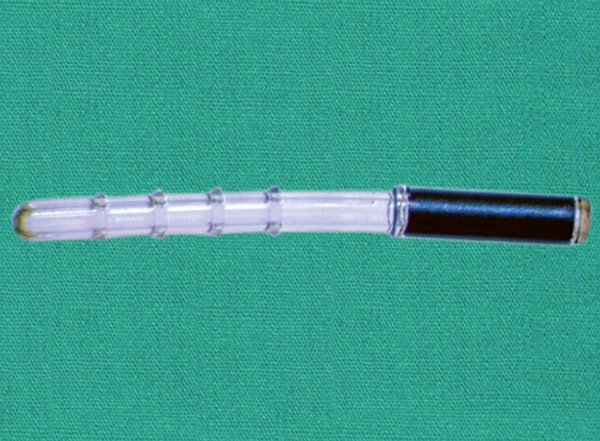
iStent Supra

*Device:* The iStent Supra (Glaukos, corporation) is made of polyethersulfone and titanium^37^ (Fig. 7). An investi-gational device exemption study is currently taking place and the company projects the FDA approval in the United States by 2018.^38^ It has, however, a European CE mark.

*Surgical technique:* As the iStent Supra is implanted from an *ab-interno* approach, similar to the CyPass Micro-Stent, it can be placed at the time of cataract surgery. The implantation process is similar to that of the CyPass Micro-Stent.

*Clinical data:* Not much has been published regarding the efficacy and safety of this product yet. One study is available after a 12-month follow-up.^22^ Seventy-three patients underwent surgery and 42 completed the whole trial. The mean IOP before surgery was 20.4 mm Hg ± 4.8 medicated and 24.8 mm Hg ± 3.4 unmedicated. At 1 year after implantation and medical treatment by Travoprost, the mean IOP reached 13.2 mm Hg. Therefore, 98% of the eyes (all but one) were considered as success (reduction in IOP of ≥ 20% with reduction in one medication). In the remaining eye, the IOP was 18 mm Hg, with reduction in one medication. Furthermore, 90% reached an IOP < 15 mm Hg with reduction in one medication. No major complications were found.

### Ab-externo

Gold Implant

**Fig. 8:**
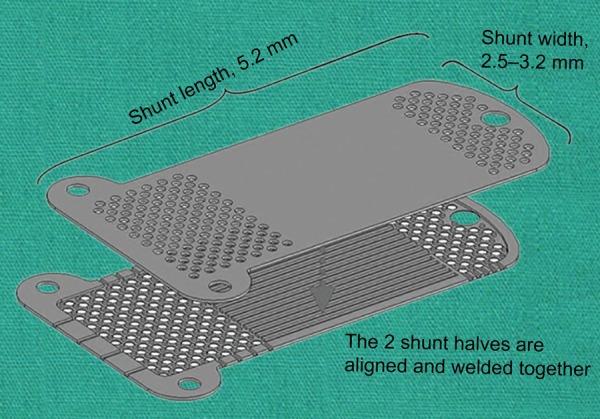
SOLX Gold Shunt

*Device:* The SOLX Gold Shunt (SOLX, Inc.) is a 24-karat gold rectangle-shaped implant composed by two plates^23-26^ (Fig. 8). The first generation of the implant came with a dimension of 5.2 × 3.2 × 44 μm^23,25,27^ and the current generation displays a thickness of 68 μm.^27^

Two rounded projections on the suprachoroidal end ensure the stability of the implant, by anchoring in the suprachoroidal space.^23,24^ Squeezed between the two golden plates, 19 channels (9 open, 10 closed) allow the aqueous humor to flow from the anterior chamber end to the suprachoroidal end. Ingress of aqueous humor from the anterior chamber is permitted owing to 60 holes 100 μm in diameter and one bigger hole 300 μm in diameter. In the same fashion, the posterior end allows efflux of liquid into the suprachoroidal space with 117 holes 110 μm in diameter. Both ends harbor additional lateral channels.^23^

It has been CE-marked since 2005, but is still an investigational device in the United States.^39^

**Fig. 9:**
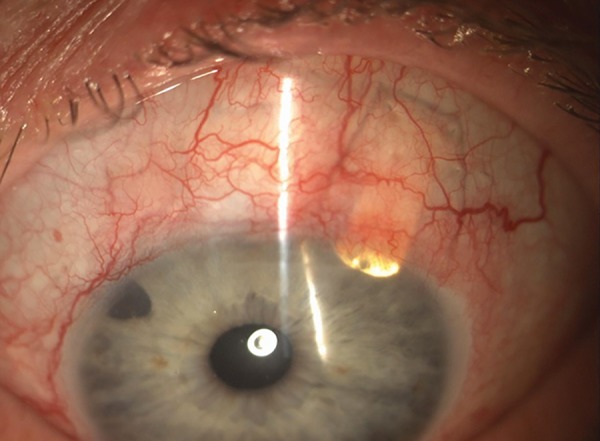
Slit-lamp examination of an eye with the SOLX Gold Shunt

*Surgical technique:* The surgical steps have been described by Melamed et al.^23^ The operation takes place under local anesthesia. The globe is to be maintained still using either a bridle suture around the superior rectus muscle, or a corneal traction suture. A fornix-based conjunctival flap is fashioned, followed by cauterization of episcleral vessels. Two millimeter posterior to the limbus, a full-thickness scleral incision is performed to expose the supraciliary space. The anterior chamber is then entered with a crescent knife at a plane of 90% scleral thickness. Further posterior dissection is proceeded for 2 mm using the same crescent knife or a blunt spatula. Then the gold shunt can be introduced with its anterior portion placed in the anterior chamber and its posterior portion in the supraciliary space. The implant must be positioned such that 1 to 1.5 mm of its length are visible in the anterior chamber (Fig. 9). Finally, the sclera and the conjunctiva are closed with 10-0 nylon sutures.

*Clinical data:* Evidence is equivocal concerning the efficacy of this device. Although several clinical trials concluded with a high success rate of the Gold Shunt^23,25,28,29^ a retrospective study by Hueber et al^27^ showed less promising results.

The first pilot study was conducted by Melamed et al in 2009 on 38 patients.^23^ At the 12-month follow-up, the mean IOP was 18.2 ± 4.6 mm Hg (compared to 28.8 ± 3.9 before surgery) and the mean number of IOP-lowering medication was 2.0 ± 0.8 (compared to 1.5 ± 1.0 before surgery). The surgical success rate was 79% (30 patients), defined as an IOP between 5 and 22 mm Hg. Complete success was reached in 13.2% (5 patients), defined as an IOP between 5 and 22 mm Hg and the absence of IOP-lowering medication. The most common complications were mild to moderate hyphema (21%, 8 patients).

Mastropasqua et al have reported a case study of 14 patients.^28^ At the end of the follow-up (15.4 ± 5.4 months), they found a postoperative mean IOP of 22.1 ± 10.6 mm Hg (compared to 28.8 ± 3.9 preoperatively) and no statistically significant decrease in IOP-lowering medication. Successful implantation was achieved in 57% (8 patients), defined as a decrease in IOP of 30% or more. Complications were not reported.

In a prospective uncontrolled case series study, Figus et al studied 55 patients who underwent gold shunt implantation.^25^ After 2 years of follow-up, they obtained a mean IOP of 13.7 ± 2.98 (compared to 30.8 ± 8.8 preoperatively) and a mean number of IOP-lowering medication of 1.55 (compared to 2.13 ± 1.61 preoperatively). A total of 67.3% (37 patients) met the criteria for qualified success (IOP < 21 mm Hg and a reduction of at least 33%) and 5.5 (3 patients) for complete success (IOP < 21 mm Hg and a reduction of at least 33%, without any IOP-lowering medications). No serious complications were reported and the most common ones were mild to moderate hyphema (12 patients).

In a prospective randomized clinical trial, Skaat et al compared the efficacy and safety of the gold shunt with the Ahmed glaucoma valve.^29^ Two models of the Gold Shunt were tested, one of 24 μm of micro-channel diameter and one of 48 μm. The follow-up period was 49.4 ± 5.2 months and 40.7 ± 4.4 months for the 24 and 48 models respectively. The mean IOP for the 24 Gold Shunt was 20.0 ± 1.9 mm Hg (compared to 25.7 ± 0.7 mm Hg preoperatively) and the mean number of medication went from 2.9 ± 0.6 to 1.7 ± 0.8. For the 48 model, it was 17.9 ± 2.3 mm Hg (compared to 35.6 ± 2.2 mm Hg preoperatively) and the mean number of medication went from 3.2 ± 0.5 to 1.8 ± 0.4. The success rate, defined as an IOP between 5 and 22 mm Hg and a decrease of 20% or more, was achieved in 77.8% in the 24 μm Gold Shunt group and 72.7% in the 48 μm model. Overall, they found that the Gold Shunt and the Ahmed valve showed similar efficacy. There was no serious complication.

In Hueber et al’s clinical trial, 31 patients implanted with the Gold Shunt were analyzed up to 4 years postoperatively.^27^ Nearly all (97%) of their patients did not meet the success criteria, meaning an IOP <21 and >5 mm Hg with a reduction of at least 20%. The mean IOP went from 26.58 ± 10.14 mm Hg before implantation to 27.19 ± 10.44 mm Hg after. Although they mention a slightly different surgical technique and a stricter definition of success they cannot explain the huge difference with the previous studies.^27^ They also reported serious complications, such as retinal detachment, endophthalmitis, suprachoroidal hemorrhage, low grade inflammation and rubeosis iridis.

In order to have a better understanding of implant failure mechanisms histological analysis^30^ as well as electron microscopy analysis have been conducted.^31^ They both showed a fibrosis reaction not only surrounding the device but also obstructing the micro-pores within the stent.

Overall, although most studies found the Gold Shunt to be both safe and efficacious, excessive tissue reaction and fibrosis have not been completely excluded.

Aquashunt

**Fig. 10:**
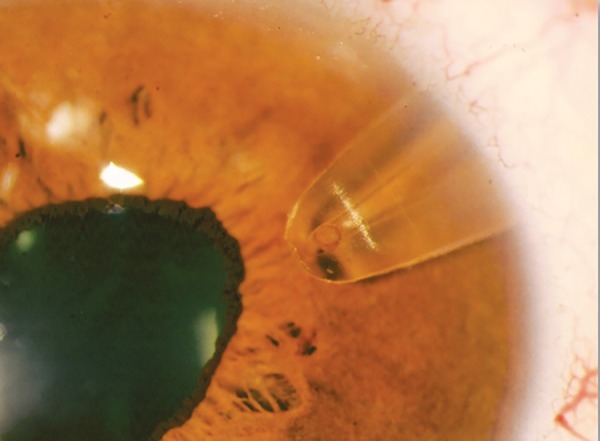
View of an eye with an implanted Aquashunt *(Courtesy:* Prof B Shields)

*Device:* The Aquashunt (OPKO Health, Inc) (Fig. 10) was primarily designed for use in less economically developed countries. As such, its concept was to match some conditions, such as relatively low cost, implantation simplicity, and low postoperative follow-up requirement.

**Fig. 11:**
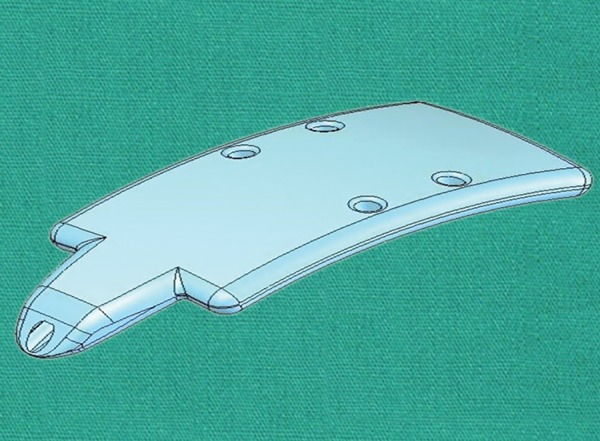
Aquashunt device *(Courtesy:* Prof B Shields)

**Fig. 12:**
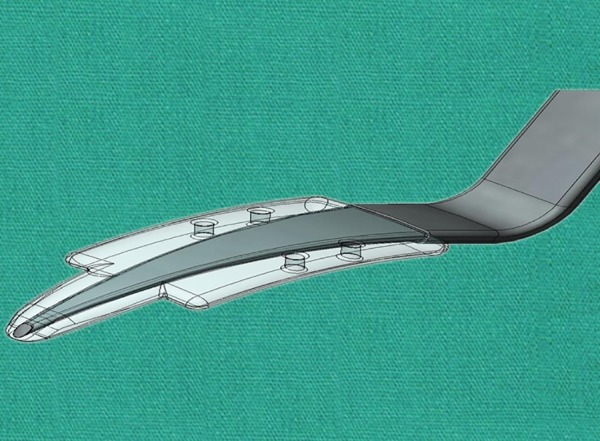
Long tapering channel within the Aquashunt device *(Courtesy:* Prof B Shields)

The device, made of polypropylene, has a dimension of 10 × 4 × 0.75 mm and is curved to accommodate the eye’s shape. The implant goes from its 4 mm width to 2 mm at a proximal extension, to 1.1 mm at the very tip (Fig. 11). This extension serves as a spatula for the implant insertion. Within the device a channel tapers longitudinally from 0.4 × 0.7 mm at the tip to 0.4 × 1.6 mm distally (Fig. 12). Finally, four islets (two at each side of the channel) provide anchoring sites for sutures to the sclera.

*Surgical technique:* A conjunctival incision is fashioned approximately 5 mm posterior to the limbus. Then a full thickness 5 mm corneal incision is performed. The insertion tool on which the Aquashunt is mounted is used similarly to a cyclodialysis spatula to dissect the suprachoroidal space to reach the anterior chamber. When the proximal extension has fully reached the anterior chamber, the shoulders of the device prevent further implantation and the guide can be removed. The implant is then secured with sutures and the scleral and conjunctival incisions can be closed.

*Clinical data:* The first clinical studies have been launched in 20 09.^40^ Fifteen patients underwent Aquashunt implantation in two centers. Prior to operation all patients had uncontrolled IOP. After the 12-month follow-up, explantation of the device was needed in one case due to pain. In terms of pressure reduction, there were three cases of unsatisfactory pressure reduction, while three others displayed on the contrary hypotony. However, the other eight patients showed an IOP reduction of 31% (13-46%). From these, four achieved satisfactory pressures with concurrent medical therapy.

STARflo

*Device:* The STARflo Glaucoma Implant (iSTAR Medical SA) is a silicon device designed to be implanted partially in the suprachoroidal space and intrascleraly, draining aqueous humor through its micropores. It has a length of 8 mm, width of 5 mm (3 mm at the neck), and a thickness of 275 μm. The STARflo Glaucoma Implant is 100% made of the STAR^R^; biomaterial, derived from NuSil med-6215 (a silicone elastomer), which is proved to have met the standards for long-term biocompatibility. The device has been CE-marked in Europe but is still an investigational device in the United States.

**Fig. 13:**
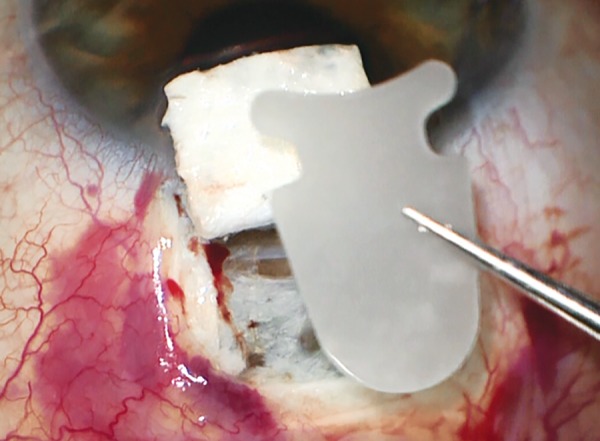
Scleral dissection from the choroid posteriorly for the implant

**Fig. 14:**
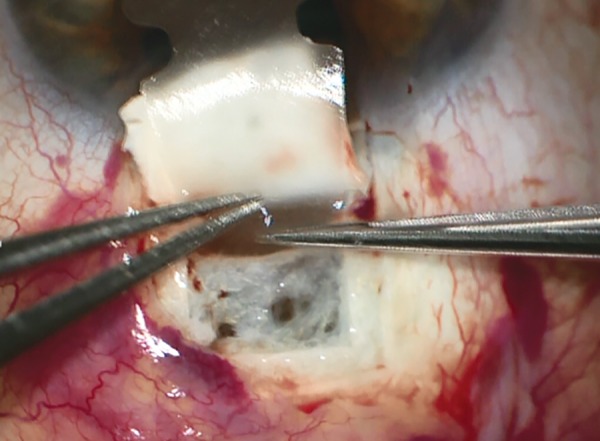
Insertion of implant into the suprachoroidal space

*Surgical technique:* Under local anesthesia, a fornix based conjunctival flap is created, followed by hemostasis of episcleral vessels. A rectangular (6-7 mm wide, 3 mm long) superficial scleral flap (50% depth) is created. Then the choroid is reached with a 5 to 6 mm incision in the second sclera layer, with a remaining surrounding scleral bridge of 1 to 2 mm. The anterior chamber is then reached with a 3 mm wide incision through the trabecular meshwork. To allow implantation of the device, the sclera is also dissected from the choroid posteriorly with a blunt spatula. The posterior half of the implant can now be introduced through the 5 to 6 mm incision into the suprachoroidal space (Figs 13 and 14). After that, the head of the implant can be inserted into the anterior chamber through the 3 mm incision. Once the implant in place, the scleral flap can be sutured, as well as the conjunctival flap.

*Clinical data:* As the implant is still relatively new, few clinical trials exist attesting of its efficacy and safety. In a 12-month clinical trial with the first implanted patients, data of three patients were collected.^32^ The mean IOP at 12 months was 14.3 mm Hg (compared to 37.0 mm Hg preoperatively) and the mean number of medication was 1.5 per day (compared to 3.25 per day). No adverse events were noted and postoperative complications included transient hypotony, transient choroidal hemorrhage, and transient abnormal macula.

## CONCLUSION

The advantages of suprachoroidal filtration implants are theoretically clear. First and foremost, since they are blebless, they avoid every subconjunctival filtration bleb-related complications. Such complications include hypotony, leakage, bleb failure, bleb-related infection (in short-term and in long-term), and discomfort with foreign body sensation or pain.^33^ Another advantage concerns the *ab-interno* approach more specifically, as it allows to spare the conjunctiva, reducing infections and preserving its structural integrity. In clinical trials, this seems to be confirmed by the overall positive results found for most devices in terms of safety and adverse events.

However, implants placed in the suprachoroidal space do not escape tissue reaction and implants failure, through fibrosis, can happen, as it was shown with the Gold implant.^30^ While an Nd:YAG laser goniopuncture can be performed to correct subconjunctival bleb failure.^34^ No equivalent procedure exists for suprachoroidal implant failure. The challenge lies in prevention by using materials inducing minimal tissue reaction and scarring.

Currently, the lack of long-term data makes it difficult to confirm actual clinical benefits and we would need more studies, including larger and multi-centered trials. Therefore, in our opinion it is still too early to claim superiority of suprachoroidal implants over more traditional methods, such as subconjunctival drainage implants or deep sclerectomy. However, we are indeed observing the emergence of promising and novel techniques in the field of glaucoma surgery.
